# The city and the virus

**DOI:** 10.1177/00420980211058383

**Published:** 2021-12-17

**Authors:** Max Nathan

**Affiliations:** University College London and Centre for Economic Performance, UK

**Keywords:** 城市, 冠状病毒, 不平等, 劳动力市场, 城市治理

## Abstract

Cities around the world are the epicentres of the coronavirus pandemic: both in the first wave, as the disease spread from East Asia, and now, as many countries enter a third wave of infections. These spatial patterns are still far from properly understood, though there is no shortage of possible explanations. I set out the emerging theories about cities’ role in the spread of coronavirus, testing these against existing studies and new analysis for English conurbations, cities and towns. Both reveal an urbanised public health crisis, in which vulnerabilities and health impacts track (a) urban structural inequalities, and (b) wider weaknesses in institutions, their capabilities and leaders. I then turn to ‘post-pandemic’ visions of future cities. I argue that this framing is unhelpful: even with mass vaccination, COVID-19 is likely to remain one of many globalised endemic diseases. Instead, ‘pandemic-resilient’ urban places will require improved economic, social and physical infrastructure, alongside better public policy. Describing such future cities is still highly speculative: I identify five zones of change.

## 摘要

世界各地的城市都是冠状病毒大流行的中心：无论是在第一波疫情从东亚开始传播时，还是现在许多国家进入第三波感染。尽管不乏可能的解释，但这些空间模式仍远未得到正确理解。我阐述了关于城市在冠状病毒传播中的作用的一些新理论，并根据现有研究和针对英国大都市和城镇的新分析对这些理论进行了检验。两者都揭示了城市化的公共卫生危机，其中脆弱性和健康影响与以下因素相伴随：(a) 城市结构性不平等；以及 (b) 机构、其能力和领导者的更广泛弱点。接着，我转向未来城市的后疫情时代愿景。我认为这种框架是无益的：即使进行了大规模疫苗接种，新冠肺炎仍然可能会是许多全球化的地方病之一。相反，具有“抗流行病能力”的城市场所将需要经过改良的经济、社会和物质基础设施，以及更好的公共政策。对这样的未来城市的描述仍然具有高度的推测性：我确定了五个变革区域。

Cities around the world are the epicentres of the coronavirus pandemic: both in the first wave, as the disease spread from East Asia, and now, as many countries enter a third wave of infections. This urban footprint remains far from fully understood, but there is no shortage of possible explanations. The catastrophe of the novel coronavirus^1^ has triggered a parallel wave of research and analysis. Even in May 2020, scientists had published over 7500 articles on COVID-19 (Yong, 2020), and economists had written over 110 working papers (Amano-Patiño et al., 2020) plus a book (Gans, 2020). Urbanists have also been busy, putting out hundreds of rapid-response pieces and academic commentaries, many in Special Issues like this one.^2^

In this piece I draw these strands together. I lay out competing theories about cities’ role in the pandemic, testing these against existing evidence and new analysis for English cities, towns and rural areas. The picture that begins to emerge – from England and elsewhere – is of an urbanised public health crisis in which vulnerabilities and health impacts closely track both underlying structural inequalities, and wider weaknesses in institutions, their resources and capabilities.

I then turn to ‘post-pandemic’ visions of future cities. I argue that this framing is unhelpful, not least because COVID-19 is likely to be one of many endemic globalised diseases. Rather we need to focus on ‘pandemic-resilient’ urban places, and develop economic, social, physical and governance systems accordingly. This is both a deeply speculative agenda and one which requires urgent intervention. I lay out five zones of change.

## Into the upside down

Physical proximity and face-to-face contact are central to urban living, and to cities’ success as social organisms. One of the more unsettling aspects of living through the pandemic has been the sense that proximity is now at best a friction, at worst a risk. During the first wave, lockdowns dramatically thinned out urban life; at the time of writing – August 2021 – the third wave means that city dwellers remain cautious, even in largely vaccinated countries. In parallel, the awed tone of much early commentary on COVID-19 and cities has given way to a mixture of resignation, frustration and anger – as urban life refuses to return to normality. The mental health toll of pandemic life, particularly for the young, women, minority groups and front-line workers, is now familiar: see British Academy (2021) for a review.

The science of COVID-19 is also better understood. Cevik et al. (2021) summarise the epidemiological evidence, highlighting four factors that drive vulnerability. First, host characteristics: COVID-19 is most severe for those over 60, for men and for people with pre-existing conditions. Second, contact pattern: the virus spreads through sustained close contact, especially up to five days after infection. Those living with an infected person (and unable to isolate at home) are especially vulnerable; large gatherings, especially eating together, are also risky. Third, environment: indoor transmission risk is almost 19 times higher than outdoors. Crowded indoor environments with poor ventilation pose maximum risk. These characteristics help explain why care homes, prisons, homeless shelters, student halls of residence and factories (especially temperature-controlled environments like meat-packing) are particular vectors of transmission. Most cases are spread by a small number of people; superspreading events typically involve large numbers of people in close indoor proximity, such as choir practices, weddings, nightclubs, cruises or large sporting events (Kochańczyk et al., 2020). This makes the virus very different from flu, but similar to the SARS pandemic, where over 70% of infections were linked to superspreading events.

The fourth factor highlighted by Cevik and co-authors is socio-economic: deprived households have a higher risk of exposure and worse outcomes when infected. People in poorer households are more likely to have poor existing health; they may also be more exposed at work, with jobs requiring close physical proximity and which may be classed as ‘essential’; and more exposed at home, with crowded conditions making it harder to socially distance.

## Why have cities borne the brunt?

These microfoundations highlight multiple ways in which cities and their citizens may be more vulnerable to coronavirus. So far, three broad accounts link cities and COVID-19, each emphasising different aspects of urban form and function.

One set of theories highlights how urban density and interaction diffuse disease, especially via superspreading events. Just as agglomeration economies scale with city size, so may bigger cities’ vulnerability to pandemics (Batty, 2020; Florida and Mellander, 2020; Glaeser, 2020; Stier et al., 2020), at least in the early stages (Adler et al., 2020; Carozzi et al., 2020). Big cities and pandemics have a long history (Florida et al., 2021; Glaeser, 2020); but more developed countries have largely forgotten about these urban public health challenges (Batty, 2020).^3^

A variant of this story emphasises international connectivity (Bourdin et al., 2021; Mazzoli et al., 2020; Verdell, 2020). In this account, globalisation breeds contagion via international business travel and tourism (Antràs et al., 2020), or in more critical accounts, networks of globalised capitalism (Davis, 2005). This view suggests dense global cities – such as London, Paris and New York – will be worse affected than surrounding nations. It also highlights more networked diffusion (Kuebart and Stabler, 2020) through tourism hubs, such as the towns and villages in the Italian Alps that helped spread COVID-19 across Europe in the Spring of 2020.

A second set of explanations focus on specific urban features that bring people into sustained, close, indoor contact. Public transport networks – a network of crowded, indoor spaces – could be a plausible transmission mechanism (Harris, 2020a). In this view, cities built around cars should fare better than older, denser places. Crowded housing is another candidate, especially multigenerational households and those with household members working in high-contact workplaces (Almagro and Orane-Hutchinson, 2020; Almagro et al., 2020; Fenoll and Grossbard, 2020; Harris, 2020b). This view implies that cases and deaths will be higher in poorer cities, in more crowded neighbourhoods within cities and among groups over-represented in crowded housing (Almagro et al., 2020). In parallel, urban labour markets have increasingly polarised into high-wage ‘knowledge-intensive’ work and low-wage ‘frontline’ service roles (Autor, 2019). While the first set of workers have the means and choice to work at home, the second group often cannot (Adams-Prassl et al., 2020; Dingel and Neiman, 2020). These workers are then exposed both physically and economically – to the virus if they keep working, and to loss of income if they do not (Almagro et al., 2020; Mongey et al., 2020).

These frameworks place decreasing weight on urban form and structural features, versus spatialised social and labour market inequalities. The latter accounts also highlight the intersection of urban housing, work and demographics, arguing that minority ethnic groups will be disproportionately vulnerable to coronavirus – many minority ethnic groups are poorer than average, and are more likely to be in exposed work, to live in crowded homes (Cevik et al., 2021; Mathur et al., 2020; Nazroo et al., 2020) and to commute by public transport (Almagro et al., 2020).

A third set of theories shift away from urban characteristics altogether, instead emphasising institutions and political leadership (or the lack of it). Recent experience of recent previous pandemics, such as SARS, is likely to shape institutional responses to COVID-19 (Ru et al., 2021). Any systemic weaknesses of national-level governance will amplify poor choices by individual leaders (Gaskell et al., 2020). Given a pandemic’s networked nature, more decentralised health systems might have better public health responses, since – in theory – subnational governments have better local information and can respond faster to changes on the ground. However, this crucially depends on subnational governments’ capacity and resources, which vary widely across countries (Rodríguez-Pose and Burlina, 2021). Since 2008, austerity in many countries has reduced institutional resources, making it harder to adjust to sudden spikes in demand, most notably for healthcare (Cook, 2020; Mazzucato and Kattel, 2020). Austerity may also hobble local government capacity to develop effective public health responses, especially in centralised states like the UK (Dostal, 2020; Kibasi, 2020).

These accounts are hard to disentangle empirically. The all-encompassing nature of a pandemic makes it hard to see causal links: as Kolko (2020) puts it, the patterns may be clear but not the reasons. More seriously, the overlapping nature of these explanations makes it hard to pick out specific features that aren’t closely related to others. And as outbreaks move through space, people also change their behaviour, and these responses are likely to be bigger both in cities with more cases and in those where more work can be done from home.

## On the ground: The experience of English towns and cities

I test these theories by exploring the pandemic’s spread across towns and cities in England – one of the world’s worst-hit countries. I use Public Health England data on confirmed COVID-19 cases from 1 January 2020 to 31 July 2021.^4^ While this is the best available case data, it has important limitations: in particular, it undercounts true case rates in early 2020.^5^ I combine this with information on population density from 2019 ONS Mid-Year Population Estimates, and area deprivation from the 2019 English Index of Multiple Deprivation (IMD).^6^ I work at local authority level, aggregating these to English Combined Authorities as proxies for the main conurbations or ‘city-regions’: London, Birmingham, Bristol, Leeds, Liverpool, Manchester, Newcastle-Gateshead, Sheffield and the Tees Valley.^7^ Using an ONS typology, I group other areas into ‘largely urban’, ‘mixed’ and ‘largely rural’ categories, reflecting smaller cities, large towns and small towns / rural areas respectively.^8^

**Figure 1.**
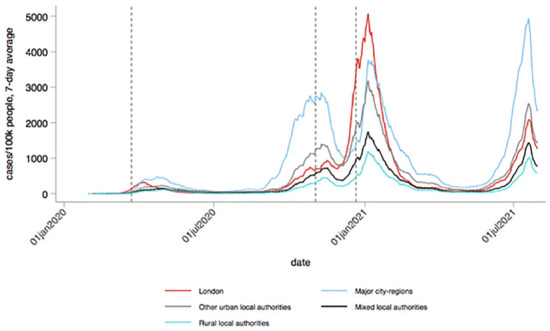
An urbanised pandemic? Weekly rolling average of new cases by area type, 1 January 2020–31 July 2021. *Source*: Public Health England, ONS. Dotted lines indicate 23 March 2020 lockdown, 5 November 2020 Tiers 1–3 introduced, 21 December 2020 Tier 4 introduced. City-regions defined by combined authority boundaries, the rest use ONS urban/rural typology.

Figure 1 shows rolling weekly average case rates from the start of the pandemic, in London, in other big city-regions and in other urban, mixed and rural areas. Dotted lines indicate national lockdowns.

Figure 1 highlights the urbanised nature of the pandemic in England, and the role of London and big city-regions in particular. Even taking early undercounting into account, big city-regions have always been hit first. However, while the pandemic spread from London in Wave 1, Waves 2 and 3 (so far) have originated outside the capital. More broadly, urban areas have higher case rates than mixed or rural local authorities.

This picture provides some support for our first set of theories. Nevertheless, London’s experience over time is not consistent with the ‘global city’ hypothesis, and higher case rates *outside* London and the conurbations at the start of Waves 2 and 3 also need explanation.

**Figure 2.**
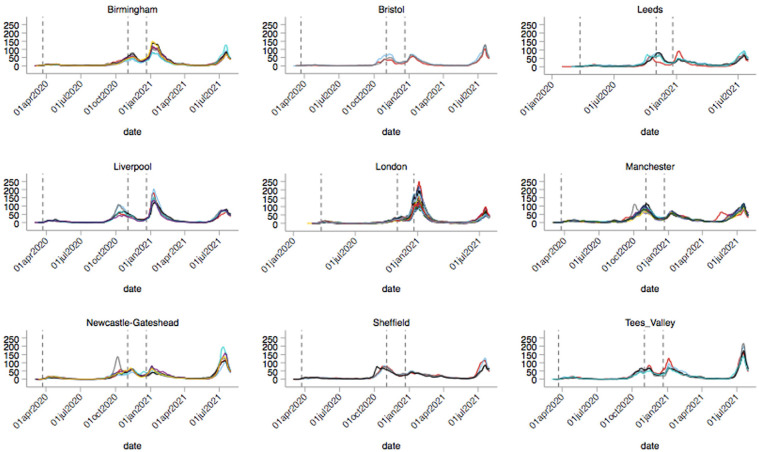
Weekly rolling average of new cases within major city-regions, 1 January 2020 to 31 July 2021. *Source*: Public Health England, ONS. Cases per 100k people, seven-day average. Dotted lines indicate 23 March 2020 lockdown, 5 November 2020 Tiers 1–3 introduced, 21 Decmber 2020 Tier 4 introduced. City-regions defined by combined authority-boundaries.

There is also substantial divergence *within* big urban areas as well as across them. Figure 2 looks further into the urban core of the pandemic, showing case rates within the big city-regions and London over time.

The first peak of Wave 2 is driven by specific parts of Liverpool, Manchester and Newcastle-Gateshead in particular, with further outliers in the other city-regions. By the second peak, in early 2021, case rates within London drive the national picture, but with significant divergence within the capital. Birmingham and Liverpool also show high case rates and within-city-region divergence. Wave 3, in early summer 2021, shows Delta variant-driven rising cases in all city-regions, but especially in Newcastle-Gateshead and the Tees Valley. By contrast, Bristol, the richest city-region, experiences visibly smaller disparities.^9^

Where are these localised hotspots? There is some movement over time, notably in London, where high case rates broadly move from central boroughs in Wave 1 to Outer East London in Wave 2 (Tower Hamlets, Newham, Redbridge) and back to central boroughs in Wave 3. Nevertheless, in non-London conurbations some poorer, largely peripheral towns and suburbs have persistently high case rates: notably Sandwell (Birmingham city-region), Oldham and Salford (Manchester), Doncaster (Leeds), Sunderland (Newcastle-Gateshead), St Helens (Liverpool), Redcar and Stockton (Tees Valley).

This time/space divergence is striking, and runs against simple accounts that emphasise city size or connectivity. Rather, the picture better fits theories that highlight urban housing, labour market conditions and deprivation – and related difference in behavioural responses – as explanatory factors. Richer areas have higher shares of residents who can adapt to the pandemic by working remotely and are better able to isolate at home. Consistently harder-hit areas are poorer, with populations more likely to be exposed both at work and in crowded housing.^10^ That is, the changing geography of COVID-19 in England is likely driven by a range of urbanised socio-economic features, more than by urban structure itself.

**Figure 3.**
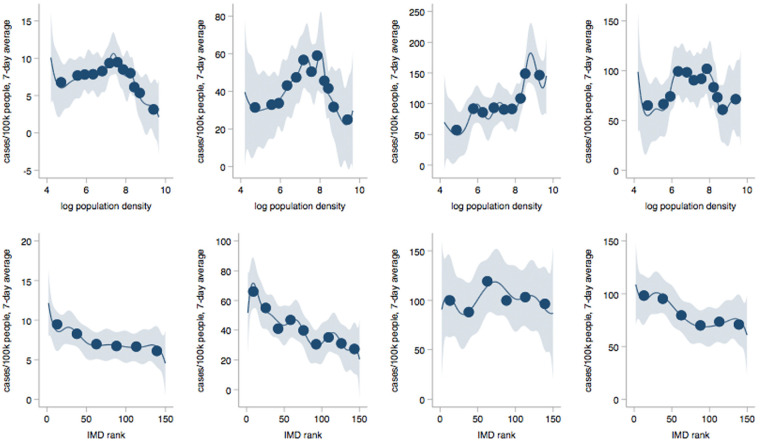
New case rates, density and deprivation at peaks of Waves 1–3. *Source*: PHE, ONS, MHCLG. *Notes*: From L.R: 29 April 2020, 13 November 2020, 5 January 2021, 19 July 2021. Binned scatterplot with robust standard errors, cubic b-spline curve and 95% confidence band. Log population density per 100,000 people. IMD Rank of average score, where 1 is most deprived.

Figure 3 summarises this line of argument. Specifically, it is a binned scatterplot showing the overall link between COVID-19 cases, population density and deprivation across all local authorities.^11^ I show the situation at the peaks of Wave 1 (29 April 2020), Wave 2 (13 November 2020, 5 January 2021) and Wave 3 to date (19 July 2021).^12^ Note that these are unconditional associations, not causal effects: many confounders are correlated with both density and deprivation.

Nevertheless, the raw picture is highly suggestive. The link between case rates and population density is lowest for the densest areas, except for the peak of Wave 2 in January 2021, when case rates peak in London. It is highest for mid-density locations – typically smaller cities and towns, or the peripheral areas of urban cores – where the case rate/density link has become stronger over time. In contrast, we can see a consistently positive link between case rates and area deprivation, which steepens between Wave 1 and Wave 2 before easing slightly (bottom panel). While the densest locations are likely to be in the biggest cities, deprivation may not follow the same geography, so that the most deprived areas in urban cores may be systematically different from those outside. I therefore re-run the analysis just for London and other conurbations. I find similar patterns: case rates are lowest in the densest big city neighbourhoods, highest in the most deprived.^13^ That is, these density and deprivation links also hold *within* the big city-regions.

Overall, these exploratory exercises suggest two main conclusions. In England to date, the pandemic is both an *urbanised* public health emergency, and one where smaller, poorer urban areas and their residents – inside and outside big cities – have borne the brunt. This picture also fits a growing body of evidence for the UK and other countries.

## Wider evidence

Globally, there is not much of a link between a country’s population density and COVID-19 cases or deaths, especially in sparsely populated nations like Sweden (Florida and Mellander, 2020). For the US, Allcott et al. (2020) suggest that urban density explains much of the variation in cases across US locations. However, Carozzi et al. (2020) show that denser US counties have earlier coronavirus outbreaks, but not higher case rates or deaths once timing is taken into account. Hamidi et al. (2020) provide similar descriptive evidence. These findings are consistent with city-level descriptive evidence for New York City, where density becomes less important over time in driving positive case share (Almagro and Orane-Hutchinson, 2020). By contrast, there is consistent evidence that urban connectivity is linked to pandemic spread (Bourdin et al., 2021; Kuebart and Stabler, 2020; Rodríguez-Pose and Burlina, 2021).

Carozzi and co-authors (2020) show that their results are partly explained by big-city dwellers who can adapt by socially distancing, most obviously by working at home. Mongey et al. (2020) show that US metro areas with larger shares of jobs that can be done remotely experienced larger drops in mobility during the first wave. They also show that more exposed workers have fewer qualifications, lower income, lower assets and are less likely to be home-owners. Alipour et al. (2021) find similar results for German localities. Similarly, the UK’s exposed workforce – including health and care sector workers, rank and file police, hairdressers, bar staff, primary and nursery teachers – is more female than male, around one in five minority ethnic (twice the population share) and has a large minority earning below median wages (Office for National Statistics, 2020).

City-level studies match this picture. In New York, zipcodes with the sharpest reductions in trips saw the smallest number of COVID-19 cases (Glaeser et al., 2020). These were typically high-income neighbourhoods in the city, with smaller numbers of essential workers or public transport users. Almagro et al. (2020) concur, finding that commuting to and from poorer neighbourhoods is strongly linked to case rates and hospitalisation in New York’s first wave.

Evidence on housing is sparser. Fenoll and Grossbard (2020) show that COVID-19 deaths are higher in countries or states with higher rates of intergenerational residence. For London, Harris (2020b) finds a positive link between large household size and coronavirus deaths in March–April 2020; for New York, household crowding is also linked to COVID-19 spread, especially for poorer and minority ethnic households (Almagro and Orane-Hutchinson, 2020).

Several studies show links between these vectors of exposure and socio-demographic characteristics. For the UK, Mathur et al. (2020) show clear positive links between social inequality and minority ethnic status, and COVID-19 case rates, intensive care admission and mortality. Alsan et al. (2021) demonstrate similar health inequalities in the US. Harris (2020b) and Nazroo et al. (2020) document outsize risks for minority ethnic groups in London. For New York, Almagro et al. (2020) show that zipcode-level household and work exposure is linked to lower income and higher non-white population share.

Evidence on institutions is much sparser. Strong national institutions *and* recent pandemic experience appear key. At least in the EU, national government quality has a much larger link to Wave 1 excess mortality than regional government quality or autonomy (Rodríguez-Pose and Burlina, 2021); even in highly devolved countries such as Switzerland, federal institutions have played central roles (Willi et al., 2020). In countries and cities that had experienced SARS in 2003 – whether East Asian or not – policymakers were faster in rolling out policy responses to COVID-19, and individuals were more compliant with social distancing than non-SARS comparators (Ru et al., 2021). Apart from Canada, European and North American countries, among others, lack such experience. On top of this, differences in leadership quality also matter. Gaskell et al. (2020) and Shrimsley et al. (2020) trace the multiple missteps of the UK government – around lockdown, supplies of PPE, test and trace systems, public communication and co-ordination with devolved administrations in English cities, Scotland and Wales. On both institutional performance and political leadership, the UK has – until very recently – performed substantively worse than comparable developed countries. Against this picture, vaccine procurement and rollout has been a welcome success – although other countries have now caught up with the UK’s lead.

The limited powers of UK local government also mean that national errors directly affect urban outcomes. Although local authorities have established contact tracing teams, national government centralised and contracted out services to inexperienced consultants; statutory sick pay remains insufficient to allow low-paid workers to isolate; and political energy has been diverted into public battles with city-region Mayors (Williams, 2020).

Given the global and interconnected nature of the pandemic, few of these studies can make clear causal links between specific urban features and health impacts. Nevertheless, together they present a striking body of descriptive evidence. As with the English experience, the crisis that is revealed is less *urban* than *urbanised*. Vulnerabilities and health impacts closely track both underlying structural inequalities in urban areas – spatial, social, economic and demographic – and wider weaknesses in institutions and leadership.

## Post-pandemic cities?

Where might this crisis leave cities in the coming months and years? The pandemic has spawned two main strands of speculation about the urban future. One predicts ‘the end of cities’, featuring often-apocalyptic predictions about the end of megacities, triggered by a mass shift to working from home and the subsequent collapse of central business districts and their local service economies (Heath, 2020; McFarlane, 2021; O’Connor, 2020). The other focuses on the ‘post-COVID’ or ‘post-pandemic’ city (Balland, 2020; Batty, 2020; Florida et al., 2021; Kleinman, 2020), a world in which cities have survived the coronavirus, but entered a new period of altered normality: new frictions on urban mobility, interaction and everyday life (Lichfield, 2020).

Predictions of the end of cities are, in one sense, easily dealt with. Cities and pandemics have a long history (Kenny, 2021). Cities and urban systems are also broadly resilient to destruction from war (Davis and Weinstein, 2002; Hanlon, 2017), natural disasters (Boustan et al., 2020; Glaeser, 2021) and past pandemics (Francke and Korevaar, 2021; Glaeser, 2020; Kleinman, 2020). Dismissing these arguments out of hand would be a mistake, however. Major disruptions can lead to long-term changes in urban form, and some impacts can be lasting, both in terms of persistent economic disparities within cities (Ambrus et al., 2020) and upward shifts in national inequality (Furceri et al., 2020).

A bigger issue, often overlooked, is the nature of ‘pandemic risk’ (Glaeser, 2020). This reflects both the chances of future pandemics, and when (and how) different countries overcome this one. The higher the first, and the longer the second, the greater the disruption to urban systems, places and communities (Florida et al., 2021; Glaeser, 2020; Nathan and Overman, 2020).

The novel coronavirus will very likely not be the last globalised pandemic: COVID-19 is merely the first of many recent candidates – including SARS, MERS, avian flu, Ebola and H1N1 – to go global (Davis, 2005; Kenny, 2021). The more this is understood, the more likely it is to produce shifts in social and economic life, especially if people believe risks have not been mitigated: Florida et al. (2021) evocatively name this ‘social scarring’. Such shifts may also occur during the current pandemic, as uneven vaccine rollouts battle new COVID variants. Nathan and Overman (2020) argue that a longer exit increases the chances that current forced experiments become norms (e.g. in home working and online shopping), not least through improved online tools (e.g. for mass communication and interaction). It may thus create new collective equilibria even though the earlier fundamentals remain. If everyone who can is now working from home, for example, the benefits of urban proximity are not accessible – even if these still exist in principle.

These dynamics will differ across nations (and within them). We now know that individual changes in behaviour in this pandemic have been primarily driven by fear of infection (Allcott et al., 2020; Andersen et al., 2020; Bundorf et al., 2021; Dave et al., 2021; Elenev et al., 2021; Goolsbee and Syverson, 2020). Countries and cities with effective vaccination programmes and wider public health policies – plus public confidence in them – may thus return faster to some kind of ‘normality’, even if COVID-19 remains endemic. Here, changes to urban life will largely involve voluntary experimentation, alongside new frictions from virus management and adapting to future pandemic risk.^14^ By contrast, in countries where the policy response is poor, mass lockdowns and forced adaptation will continue, with all the difficulties that entails. That is, we will likely see multiple, overlapping urban worlds, with cities moving between more or less friction/constraint as viruses arise and mutate, and policy responds.

Disparities in vaccine rollout give a sense of how these shifting geographies might work. At the time of writing, there are huge differences in vaccination rates both across and within nations. Cross-country disparities imply big variations in required policy responses, with greater reliance on lockdowns, masks and distancing versus vaccines and unlocking. But if less vaccinated countries act as ‘variant factories’ – as in the Delta mutation – this may undermine policy in more vaccinated locations.

Within-country disparities may generate similar dynamics. At the time of writing, complete vaccine coverage in England varied from over 75% in rural areas with older populations, such as Dorset, to under 40% in poorer, more diverse London neighbourhoods like Newham, which also have younger populations. While coverage is still growing, it is notably worse in London and other conurbations. More worryingly, high case rate and low take-up geographies overlap, with lower vaccine coverage in the densest and most deprived local authorities, including those within conurbations.^15^

All of this suggests that generalised ‘post-pandemic’ or ‘post-COVID’ framings of urban futures are unhelpful. The task for applied urban researchers and policymakers is ‘pandemic-proofing cities’ (Parnell, 2020), and exploring what pandemic-resilient urban life might look like.

One zone of change is public health – and social policy. Glaeser (2020) suggests that countries should spend ‘whatever it takes’ to reduce future pandemic risk. Kenny (2021) highlights four main elements: better human and animal sanitation; improved disease surveillance systems; robust test/trace/isolation protocols; and more research on tests, treatments and vaccines. Mass vaccination reduces both health risks and virus transmission (Richterman et al., 2021). On top of this, social distancing followed by robust testing, tracing and isolation can keep new variants in check while allowing economic and social life to re-open. This requires mass testing with rapid turnaround, plus immediate isolation for symptomatic people and effective contact tracing (Aleta et al., 2020; Cevik et al., 2021). It also requires income protection to allow low-wage people to self-isolate, and protective systems in workplaces. Such systems have proven effective for previous airborne pandemics.

A second, more speculative zone is the future of office work, a mainstay of post-industrial cities. While home working is increasingly feasible for complex ‘knowledge-intensive’ tasks (Clancy, 2020), in most countries only a minority of jobs can be done at home (Dingel and Neiman, 2020) and this issue is more severe in less developed countries (Garrote Sanchez et al., 2020). In the UK, regular remote working rose from 6% pre-pandemic to 43% by the end of June 2020 (Felstead and Reuschke, 2020).

Will this mass forced experiment become a new norm? The costs and benefits of remote working are still poorly understood. As Clancy notes, even if remote working is not as effective as face to face, firms may trade this off for cost savings. Pre-pandemic studies suggest that shifting existing workforces to remote working leaves them as effective as before, or better (Bloom et al., 2015; Choudhury et al., 2021); but evidence from 2020 is more pessimistic (Bao et al., 2021; Gibbs et al., 2021). We also have little idea how online tools work for inducting new team members, developing new collaborations, serendipitous interaction or discovering new ideas: all things big cities continue to do well, even as the cost of travel and organising remotely has fallen. While continued mass remote or hybrid working may generate new mass workarounds for face-to-face interaction (Page, 2020), the current technological frontier still lags behind (Ayyangar, 2021).

The political economy of remote working is also unclear. Remote working passes some costs and risks from firms to workers, and shifts the domestic division of labour: even controlling for industry and occupation, women can do fewer tasks from home (Adams-Prassl et al., 2020). More broadly, while working from home remains very popular among most office workers, employers currently want significantly less of it than staff do (Barrero et al., 2021; Haskel, 2021). As firms are likely to win this battle, an immediate remote revolution now seems unlikely.

The urban geography of these changes is thus hard to predict. Firms will experiment with hybrid working, and change organisational practices around this. In any given city, a major shift to home/hybrid working will reduce overall demand for CBD space; a partial shift might actually increase it, if office work still requires social distancing (Nathan and Overman, 2020). Across cities, however, this could lead to a further concentration of ‘front office’ knowledge and business services in a few large urban cores, while more ‘back office’ activities move to the peripheries or to smaller, cheaper locations.

Third, adjustments in office work will change urban housing markets, and in geographies of local retail and leisure. As higher-paid jobs are more amenable to home working, we would expect relocation to reinforce spatial segregation (Nathan and Overman, 2020). So far we have largely seen demand shifts within cities, not out of them (Cheshire et al., 2021; Gupta et al., 2021; Judge and Pacitti, 2021; Kolko et al., 2021; Liu and Tang, 2021; Patino et al., 2021; Ramani and Bloom, 2021). Movers are relocating from urban cores to peripheries, and from smaller to larger properties. As predicted, these two effects together are pushing property prices up.

The key unknowns for local services – in particular, CBDs’ lunchtime and evening economies – derive from shifts in the demand for office space, and how far consumer demand simply shifts location from city centres to residential neighbourhoods. Some shopping will shift to local businesses; other activity will move and stay online. Lockdown has seen many households substitute online shopping for physical retail (Relihan et al., 2020). Offsetting this is reduced expenditure on commuting and clothing; against that, higher spending on office equipment and energy at home. Cicala (2020) suggests that increases in domestic energy consumption under lockdown cancelled out savings from offices and commercial spaces. This raises deeper questions about the environmental sustainability of re-organised urban economic and social systems.

A fourth zone of change is the adaptation of urban infrastructures and built form. Cities around the world have embraced forms of ‘emergency urbanism’ (Florida et al., 2021) in public spaces and public transport systems, such as wayfinding, capacity monitoring, touchless interaction and physical divisions. City leaders have also actively developed cycleways and pedestrianisation as part of a longer-term move towards ‘15-minute city’ models (OECD, 2020). However, while these modes of transport have become more popular, car use has also risen substantively. As Kleinman (2020) argues, this dynamic creates a new imperative for congestion charging and road pricing systems, at the same time as it raises new fiscal risks for city governments. The underlying contradiction can only be resolved by addressing pandemic risk – most obviously through public health measures. Longer-term shifts in the demand for urbanness – especially in big cities and city centres – will open up further questions about how office and commercial spaces could be re-used, as well as re-designing the surrounding urban grain.

A final and more difficult set of questions surround pandemic-resilient urban government. The pandemic has arguably shown the virtues of central government systems. However, one legacy of *centralised* systems is atrophied local government, which tends to lack capacity and experience to take on new roles. The stop-start, negotiated nature of recent devolution in England, combined with years of austerity, means that few local administrations may be ready to take on the array of actions outlined by (e.g.) Mazzucato and Kattel (2020) or Parnell (2020: 1114), who argues for ‘well-informed and effective government that has credibility, capacity and good information at the local scale’. How UK cities might (re)build and deploy these capacities remains a profound challenge. Part of the answer will be allowing cities to deploy resources they already possess (OECD, 2020). As Gaskell et al. (2020) and others suggest, a further component is substantive local commissioning powers and resources, including a larger local tax base. But it will also require a larger realisation that cities – the hot cores of the pandemic – also need to be able to take their own steps to develop into pandemic-resilient urban communities of the future.

## Notes

1. In this article, I use ‘coronavirus’ and ‘COVID-19’ interchangeably. Strictly speaking, SARS-nCoV-2 is the novel coronavirus, and COVID-19 is the disease that it causes.

2. See, among others, *Cities, Journal of Regional Science, Regional Science and Urban Economics* and *Tijdschrift voor Economische en Sociale Geografie*.

3. Balaji Srinivasan makes the point well here: https://twitter.com/balajis/status/1259483087785783297l (accessed 5 November 2020).

4. Data taken from https://coronavirus.data.gov.uk (accessed 5 August 2021). New cases indicate the number of people with a positive COVID-19 test (either lab-reported or rapid lateral flow test) on the specimen date (the date when the sample was taken from the person being tested). Cases are allocated to the person’s area of residence. I use seven-day rolling averages of new cases per 100,000 people. This approach helps smooth out spikes from misreporting or other administrative errors, such as the spreadsheet-related delay which added around 16,000 cases on 3–4 October 2020.

5. This data likely undercounts the true extent of the pandemic, especially in the first half of 2020. First, UK testing capacity has expanded greatly since the start of 2020, and case data from July 2020 shows both hospital (‘Pillar 1’) and community tests (‘Pillar 2’). Second, around one in three coronavirus cases are asymptomatic.

6. April 2019 Mid-Year Population Estimates from the UK Office of National Statistics (Office for National Statistics, n.d.a). IMD data from the Ministry of Housing, Communities and Local Government (n.d.). The IMD measures relative deprivation at small area level across seven individual domains (income, employment, education, health, crime, access to housing, physical environment), which are then summarised in the overall Index.

7. Specifically, I use Upper-Tier Local Authorities as my spatial unit and aggregations of local authorities for urban cores. Large cities contain multiple local authorities. For London I use the Greater London boundary. For other urban cores, I use Combined Authority boundaries to proxy conurbations or city-regions (taken from Office for National Statistics, n.d.b). Combined Authorities are aggregations of contiguous local authority units, agreed by local political leaders and central government and designed to cover large urban areas. Note that these are not true commuting zones or functional economic areas, like MSAs in the USA.

8. I use the ONS 2011 Rural Urban Classification, updated for 2019 local authorities in England. The broad RUC2011 typology classifies local authorities into three types: predominantly urban (43% of local authorities outside major city-regions); urban with significant rural (21%); predominantly rural (36%). For details, see Office for National Statistics (n.d.c).

9. Bristol city-region contains fewer local authorities (three) than any others, so some of this result may be mechanical, hiding bigger differences at the neighbourhood level. Nevertheless, the figure also shows substantial within-city-region divergence in Sheffield (four LAs), Leeds and Tees Valley (five LAs) as well as in London (33 LAs).

10. While cities like Manchester and Liverpool have large student populations – numbering in the tens of thousands – it is less clear that university neighbourhoods have driven city-level spread, despite localised outbreaks. See this discussion by Chris Giles and others: https://twitter.com/chrisgiles_/status/1318520979321245696?s=21 (accessed 6 November 2020).

11. Binned scatterplots visually represent the mean relationship between two variables. ‘Binning’ divides our data into equally sized groups based on the number of observations, using the mean value of each density/IMD group to plot against case rates. I add a best-fit curve and 95% confidence interval using the binsreg Stata package (Cattaneo et al., 2019). Diagnostics using a simple scatter plot with line of best fit strongly suggest that the true relationship between cases and density/deprivation is non-linear.

12. While this is not a continuous time series, it is informative about *changes* over time. I also run a sensitivity check using National Lockdown dates (23 March, 5 November, 21 December 2020). Results are very similar, and are available on request.

13. Results are available on request.

14. For example, Israel vaccinated over 80% of its population in early April 2021, but reimposed some restrictions in Summer 2021, and will conduct booster vaccinations in Autumn 2021.

15. Results available on request.
